# Introduction to the Special Issue on Healthy Start

**DOI:** 10.1007/s10995-017-2404-y

**Published:** 2017-11-30

**Authors:** Johannie G. Escarne, Hani K. Atrash, David S. de la Cruz, Benita Baker, Madelyn Reyes

**Affiliations:** Health Resources and Services Administration, Maternal and Child Health Bureau’s, Division of Healthy Start and Perinatal Services (HRSA\MCHB\DHSPS), Rockville, MD USA

This Special Issue highlights Healthy Start, one of the nation’s earliest programs designed to help reduce high rates of infant mortality in U.S. communities. Healthy Start is a federally-funded, community-driven program dedicated to reducing disparities in maternal and infant health, and a critical component of the maternal and child health safety net (SACIM 2013). Healthy Start works in communities with infant mortality rates at least 1.5 times the national average, and high rates of low birth weight, preterm birth, and maternal morbidity and mortality.

In 2016, Healthy Start celebrated its 25th year of Federal funding. Authorized by Congress in 1991, Healthy Start has grown from a demonstration program in 15 communities, to a network of 100 Healthy Start programs in 37 states and the District of Columbia (HRSA/MCHB 2017). Over its first 25 years, Healthy Start has evolved from a program focused primarily on improving pregnant women’s access to prenatal care to a developmentally focused program seeking to improve women’s and children’s health from preconception to early childhood, creating the foundation for optimal infant and young child health and development (Kotelchuck 2012). Healthy Start serves women of reproductive age, pregnant women, mothers who have just given birth, and infants and families from birth to the child’s second birthday. Healthy Start involves fathers and supports couples with reproductive life planning.

On an individual level, Healthy Start strives to ensure access to community-based, culturally sensitive, family-centered and comprehensive health and social services to women, infants, and their families. Healthy Start programs provide: referral and ongoing health care coordination for well-woman, prenatal, postpartum, and well-child care; supportive/enabling services including; outreach, case management, home visiting, father involvement, child development education and parenting support, linkage to housing assistance, adult education, and job training programs; and health education and support related to breastfeeding, safe sleep, perinatal mood disorders, substance use, and intimate partner violence.

At the community level, Healthy Start participates in community action networks (CANs) that mobilize community residents, healthcare and social service providers, as well as other local organizations to coordinate and integrate services, and steer local action to address social determinants of health related to poor birth outcomes (National Healthy Start Association (NHSA) 2010).

This Special Issue includes original research articles from Healthy Start grantees across the nation, in urban, rural, and border communities, as well as articles focusing on the program as a whole. This collection of papers showcases defining characteristics of Healthy Start, including its simultaneous focus on supporting individuals and strengthening communities, its commitment to bringing the consumer voice to efforts to improve maternal and infant health, its embrace of a community-driven systems development approach to improving local service networks, its early adoption of community health workers as part of the program team, and its use of data to guide program improvements.

Healthy Start provides a forum for the community/consumer voice, actively soliciting input from the women and families served and using this information to identify service needs and gaps. The paper by Roman et al. describes the perspectives of Healthy Start participants seeking, using and navigating prenatal, postpartum and interconception services, in order to inform community efforts to redesign services to better meet the needs of pregnant and postpartum women. Selchau et al. present case stories to illustrate barriers to achieving good pregnancy outcomes, and Healthy Start’s role in reducing these barriers. The article by Browne offers findings from focus groups with Healthy Start participants to identify service characteristics, including relational engagement with staff, that impede or enhance successful service navigation. Another paper by Selchau et al. describes factors associated with first trimester prenatal care initiation among Hispanic women along the U.S. Mexico border.

A hallmark of Healthy Start is its simultaneous focus on individuals, systems, and communities as it seeks to connect women and families to high quality services and care. The article by Leruth describes just such a multilevel approach to breastfeeding promotion, which includes both individual education and counseling, and partnership with a local hospital to provide lactation support to maternity patients, with the aim of increasing breastfeeding initiation rates. Anderson et al. present house parties as an innovative and effective, community-based model which provides outreach to women in need of services, delivers health education on a variety of MCH topics, and offers referrals. Thomas et al. describe a doula program which provides trained birth assistants to low income women, in partnership with the city Health Department and local hospitals.

While Healthy Start has emphasized the need for multi-sectoral community engagement and collaboration since its inception, in 2014 the program adopted Collective Impact as a community engagement framework for reducing perinatal health disparities and infant mortality. The paper by Bradley et al. describes the development of peer learning networks designed to accelerate the learning and experience of Healthy Start grantees in the application of Collective Impact to achieve their mission and goals.

From its earliest days as a demonstration project, Healthy Start programs have employed community health workers (CHWs) as front line staff working directly with program participants. CHWs go by many names in Healthy Start, including navigator, parent advocate, family educator, outreach worker, and other titles. The article by Raffo et al. defines the role of CHWs within a Healthy Start care coordination team, identifying core functions and collaboration with other team members such as social workers and nurses. The paper by DeAngelis et al. describes the results of a study which explored how CHWs in Healthy Start programs across the country serve participants, what roles they take on and what skills they need to have, in order to inform the development of core competencies and a standardized training program.

The final set of papers explores how Healthy Start programs have used and are using individual, program and community level data to identify opportunities for intervention, improve program performance and monitor outcomes.

The paper by Kothari describes a Perinatal Periods of Risk (PPOR) analysis which identified broad areas of risk within a Healthy Start community, and helped to isolate the effects of race and socioeconomic status on infant mortality, in order to inform a community action initiative. The article by K. Brown et al. examines the association of birth outcomes with how early in pregnancy the participant enrolled. The paper by H. Brown and Beasley describes the use of a Fetal and Infant Mortality Review (FIMR) analysis to help shape strategies for prevention of infant mortality in a Healthy Start community. Finally the article by Dwyer et al. describes the national evaluation strategy for assessing the program’s implementation, participant utilization of Healthy Start services, and the effect of the program on pregnancy outcomes.

As it enters the second half of its third decade, Healthy Start remains true to its core mission of reducing maternal and infant health disparities in high risk communities. Using the strategies and approaches described in this issue, Healthy Start will continue to work toward the goals of reducing differences in access to and use of health services, improving the quality of the local health care system, empowering women and their families, and increasing consumer and community participation in health care decisions. By supporting women and families before, during, and after pregnancy, and engaging community partners to enhance systems of care, Healthy Start aims to give the next generation a strong and healthy start in life.
